# Observational study on initiation of dapagliflozin in heart failure with reduced ejection fraction in the United Kingdom

**DOI:** 10.3389/fcvm.2026.1858743

**Published:** 2026-09-08

**Authors:** Steven Coombs, Angela Tithecott, Cristina Thiebaud, Parminder Chaggar, Diane Barker, Prithwish Banerjee, Henry Oluwasefunmi Savage, Alastair Gray, Geraint Jenkins, Mohammad Albarjas, Liz Hennessy, Joanna De Courcy, Hannah Wallis, Thom Dewar, Reece Manuell, Sophie Barlow, Samuel Adamsson Eryd, Jil Billy Mamza, Tamsin Morris

**Affiliations:** 1 Sussex Cardiac Centre, University Hospital Sussex NHS Foundation, Brighton, United Kingdom; 2 Northern Devon HealthCare NHS Trust, Northern Devon District Hospital, Devon, United Kingdom; 3 Royal Cornwall Hospitals NHS Trust, Cornwall, United Kingdom; 4 University Hospitals of North Midlands NHS Trust, Royal Stoke University Hospital, Stoke-on-Trent, United Kingdom; 5 University Hospitals Coventry and Warwickshire NHS Trust, Coventry, United Kingdom; 6 Mid and South Essex NHS Trust, Basildon University Hospital, Basildon, Essex, United Kingdom; 7 Southern Health and Social Care Trust, Craigavon Area Hospital, Craigavon, County Armagh, United Kingdom; 8 Swansea Bay University Local Health Board, Swansea, United Kingdom; 9 King’s College Hospital NHS Foundation Trust, King’s College Hospital, London, United Kingdom; 10 Adelphi Real World, Bollington, United Kingdom; 11 AstraZeneca, Biopharmaceuticals Medical, CVRM Evidence, Mölndal, Sweden; 12 AstraZeneca, Medical and Scientific Affairs, BioPharmaceuticals Medical, London, United Kingdom

**Keywords:** dapagliflozin, heart failure, longitudinal study, quality of life, United Kingdom

## Abstract

**Background:**

Sodium–glucose cotransporter-2 inhibitor (SGLT2i) dapagliflozin has shown favourable outcomes in trials for heart failure with reduced ejection fraction (HFrEF), but little is known about real-world prescribing practices, tolerability, or impact on patient quality of life (QoL) in the United Kingdom.

**Methods:**

This study was an observational longitudinal cohort study of adult patients with HFrEF in the UK, following the date of first initiation of dapagliflozin. Baseline patient characteristics, concomitant medication prescribed at enrolment, and changes to these treatments were recorded at 3, 6, 9 and 12 months following initiation. Patients completed KCCQ-23, MARS-5 and WPAI patient-reported outcome measures to assess QoL, treatment adherence and work/activity impairment, respectively, at enrolment and follow-up.

**Results:**

N = 237 patients were enrolled, with *n* = 231 remaining at study end. Mean (95% confidence interval) age was 69.3 (67.8–70.8), 70.0% were male and 91.6% White. Mean time from HFrEF diagnosis to initiation of dapagliflozin was 20.9 (15.3–26.4) months. Dapagliflozin discontinuation rate was 11.7/100 patient-years, most commonly due to adverse drug reactions (65.0%). The majority of patients received four treatment pillars at enrolment and 12-months (54.4% and 58.4% respectively). At enrolment and 12 months, 33.8% and 30.5% respectively received three out of four pillars. Mean overall KCCQ-23 scores ranged from 67.6 (64.3–70.9) to 71.6 (67.3–74.9), and patients reported high levels of adherence, with MARS-5 scores of 23.1 (22.3–23.9) or above. Overall work impairment ranged from 31.2% (18.5–43.9%) at enrolment to 16.3% (7.4–25.2%) at 12 months, and overall activity impairment from 40.5% (36.3–44.7%) to 36.8% (32.1–41.5%).

**Conclusions:**

Patients receiving dapagliflozin recorded low levels of discontinuation, suggesting high tolerability. Many patients do not receive all four recommended treatment pillars, highlighting an opportunity for further treatment optimization. Patients had fair-to-good QoL throughout the study, indicating good patient experience during HFrEF treatment including dapagliflozin.

## Introduction

Heart failure (HF) is characterized by structural and/or functional abnormalities within the heart that result in cardiac inefficiency (1). HF may be classified as HF with reduced ejection fraction (HFrEF) when the left ventricular ejection fraction (LVEF) is ≤40%, or HF with a mildly reduced (HFmrEF) or preserved ejection fraction (HFpEF), if LVEF ranges between 41%–49% or 50%, respectively (1). Between 2015 and 2019, prevalence of HF in the UK increased from 2.1% to 2.4%, while incidence increased from 4.1/1,000 to 4.9/1,000 person years, with 70.1% of 100,224 patients with phenotype data available being diagnosed with HFrEF (2).

Common symptoms of HF, including breathlessness, exercise intolerance and fatigue, may lead to a reduced quality of life (QoL), which may be improved by optimal guideline-directed HF medication (3). International guidelines recommend four pillars of treatment for HFrEF; renin–angiotensin–aldosterone system inhibitors (RAASi), beta blockers, mineralocorticoid receptor antagonists (MRA) and sodium-glucose cotransporter 2 inhibitors (SGLT2i) (4). Guidelines focus on rapid initiation and optimization of these four treatment pillars (4), following trial data showing this was a safe and efficacious approach.

The SGLT2i dapagliflozin was approved by the Food and Drug Administration in 2020 for patients with HFrEF, regardless of diabetic status (5), based on results from the phase III DAPA-HF (Dapagliflozin and Prevention of Adverse Outcomes in Heart Failure) trial. In this trial, dapagliflozin significantly reduced risks of worsening HF (hospitalization, or urgent clinic visit resulting in intravenous therapy for HF) and cardiovascular mortality by approximately 30%, and improved symptoms, physical function and QoL when prescribed in addition to optimal HF treatment (6). Dapagliflozin was approved for use in the United Kingdom's (UK) National Health Service (NHS) for HFrEF in 2021 (7), and is now approved across the ejection fraction range (8).

However, there is a lack of real-world data on the use of dapagliflozin and the impact on QoL in UK HFrEF management. Therefore, the aim of this study was to collect real-world data on the demographic and clinical characteristics of patients with HFrEF who were prescribed dapagliflozin, associated prescribing patterns, and patient perspectives on QoL.

## Methods

### Study design and population

This study was a descriptive, observational, prospective longitudinal cohort study, with retrospective baseline data collection, at 10 hospital centres in the UK (listed in Supplementary Table 1), in previously SGLT2i-naïve patients initiated on dapagliflozin for HFrEF in outpatient or inpatient settings. Patients received standard medical care as determined by the treating physician. The decision to initiate dapagliflozin was made by the treating physician during routine interactions with the patient, prior to enrolment in the study, and was not influenced by participation in the study.

The study design is shown in Supplementary Figure 1. Index date was the patient's first initiation on dapagliflozin for HFrEF between October 2021 and February 2023 and patients were enrolled in the study 14—60 days following initiation to ensure initiation was not influenced by taking part in the study. A baseline period of 12 months prior to dapagliflozin initiation was used to retrospectively collect patients’ medical history. Demographic information and clinical characteristics, as well as patient-reported outcomes, were collected at enrolment. Patients had one week to complete their patient-reported outcomes from enrolment. Changes to medical treatment, prescribing and further patient-reported outcome measures were recorded at 3, 6, 9 and 12 months following enrolment (± 1 month).

Physician-reported data were collected using electronic case report forms, and patient-reported data were collected either at site visits or remotely, using pen-and-paper or electronic questionnaires.

### Inclusion and exclusion criteria

Patients were eligible for inclusion in the study if they were:

- aged ≥18 years at study index date
- initiated on dapagliflozin for HFrEF (EF ≤40%) in accordance with product label during the index period
- provided signed and dated informed consent prior to study enrolment.

Exclusion measures were:

- being enrolled <14 days or >60 days following initiation of dapagliflozin
- having prior treatment with dapagliflozin or other SGLT2i,
- being initiated on dapagliflozin outside of local HF label
- having a diagnosis of type 1 diabetes prior to enrolment.

### Study measures

Physicians reported baseline clinical characteristics, including medical history and comorbidities, measures of heart and kidney functioning, New York Heart Association (NYHA) classification, concomitant medication use and healthcare resource utilization (HCRU). At follow-up, physicians reported changes to dapagliflozin or concomitant medication use.

Patients completed three PRO measures at enrolment and follow-up timepoints (3, 6, 9 and 12 months): the Kansas City Cardiomyopathy Questionnaire (KCCQ-23); the Medication Adherence Report Scale (MARS-5); and the Work Productivity and Activity Impairment Questionnaire (WPAI).

The KCCQ-23 is a validated 23-item questionnaire on physical limitations, symptoms (frequency, severity and recent change over time), social limitations, self-efficacy, and quality of life in the last two weeks, with all items measured on a Likert scale with 5–7 response options (9). Summary and domain scores are scaled from 0 to 100, with higher scores indicating better health status.

The MARS-5 questionnaire assesses treatment adherence, and has five questions on forgetting, changing dosage, stopping, skipping, and taking less medication, scored on 5-point Likert scales, leading to a maximum score for perfect adherence of 25 (10).

The WPAI measures impairment over the last seven days in four domains: absenteeism (work time missed), presenteeism (impairment at work), overall work impairment, and activity impairment (regular activities other than work) (11). Scores range from 0 to 100 and are expressed as impairment percentages, with higher scores indicating greater impairment.

### Statistical analysis

Data are shown as either proportion of cohort or mean, with 95% confidence intervals (CIs). The Kaplan–Meier estimator method was used to calculate time to discontinuation of dapagliflozin. Survival in the Kaplan–Meier analysis was defined as no dapagliflozin discontinuation or death. Time was taken from the start of dapagliflozin treatment to the point of discontinuation or death. Patients who did not discontinue or die during the observation period were censored at their last available observation. All statistical analyses were performed using Stata/MP (version 18.0; StataCorp LP, College Station, TX, USA).

### Ethical approval

The study received ethical approval by the North of Scotland Research Ethics Committee on 10th August 2021 and the Health Research Authority and Health and Care Research Wales on 11th August 2021 (IRAS ID: 296166, REC reference: 21/NS/0102). The study conformed to the principles outlined in the Declaration of Helsinki of 1964 and subsequent revisions.

### Patient and public involvement

There was no patient or public involvement in study design.

## Results

### Patient numbers and attrition

N = 237 patients were enrolled, *n* = 237 (100.0%), *n* = 236 (99.6%), *n* = 232 (98.3%) and *n* = 231 (97.5%) patients remained in the study at 3, 6, 9 and 12 months, respectively (Supplementary Figure 1). Patient-reported data were available for up to 229 (96.6%) patients at enrolment, and 205 (86.5% of remaining cohort), 190 (80.5%), 187 (80.6%) and 168 (72.7%) at 3, 6, 9 and 12-month follow-ups respectively, although availability of PRO data was dependent on patient completion at respective timepoints.

### Patient demographics

Patient demographic characteristics at enrolment are reported in Table 1. At dapagliflozin initiation, mean (95% CI) age was 69.3 (67.8–70.8) years. Most patients were male (70.0%) and White (91.6%).

**Table 1:** Physician-reported demographics at initiation.

	Value	95% CI
Age at dapagliflozin initiation, *n* = 237, years		
Mean	69.3	67.8–70.8
Biological sex, *n* = 237, *n* (%)		
Male	166 (70.0)	63.8–75.8
Female	71 (30.0)	24.2–36.2
Ethnicity, *n* = 237, *n* (%)		
White	217 (91.6)	87.3–94.8
Afro-Caribbean	2 (0.8)	0.1–3.0
Asian - Indian subcontinent	3 (1.3)	0.3–3.7
Asian - Other	1 (0.4)	0.0–2.3
Chinese	1 (0.4)	0.0–2.3
Middle Eastern	1 (0.4)	0.0–2.3
Mixed Race	5 (2.1)	0.7–4.9
Other	6 (2.5)	0.9–5.4
Unknown	1 (0.4)	0.0–2.3
Family history of heart failure, *n* = 237, *n* (%)		
Yes	94 (39.7)	33.4–46.2
No	68 (28.7)	23.0–34.9
Unknown	75 (31.6)	25.8–38.0
BMI, *n* = 179, kg/m^2^		
Mean	28.1	27.1–29.1
Patient-reported demographics		
Highest level of education, *n* = 230, *n* (%)		
Some secondary school	77 (33.5)	27.4–40.0
GCSE/Standard Grade	40 (17.4)	12.7–22.9
A Level/Highers	18 (7.8)	4.7–12.1
Higher education (non-degree)	45 (19.6)	14.6–25.3
University degree	21 (9.1)	5.7–13.6
Postgraduate degree or higher	12 (5.2)	2.7–8.9
Other	17 (7.4)	4.4–11.6
Employment status, *n* = 228, *n* (%)		
Employed full-time	29 (12.7)	8.7–17.8
Employed part-time	11 (4.8)	2.4–8.5
Self-employed	13 (5.7)	3.1–9.6
Unemployed	6 (2.6)	1.0–5.6
Retired (due to age)	137 (60.1)	53.4–66.5
Retired (due to ill health)	23 (10.1)	6.5–14.8
Student	0 (0.0)	0.0–1.6
Other	9 (3.9)	1.8–7.4

95% CI, 95% confidence interval; BMI, body mass index; GCSE, General Certificate of Secondary Education.

### Clinical characteristics

Physician-reported clinical characteristics at dapagliflozin initiation are shown in Table 2. Dapagliflozin was initiated at a mean of 20.9 (15.3–26.4) months following HFrEF diagnosis. In the 12 months prior to initiation, 21.5% of patients were recorded as having a HF-related hospitalization, the most recent of which was a mean of 109.5 (77.9–141.1) days prior to initiation. The principal cause of HF was unknown for 41.4% of patients, non-ischemic for 30.8% and ischemic for 27.8%. Patients were most commonly NYHA class II (49.4%), with a mean left ventricular ejection fraction of 28.1% (26.9–29.3%). Commonly recorded comorbidities included atrial fibrillation (32.5%), hypertension (29.5%) and myocardial infarction (22.8%).

**Table 2:** Physician-reported clinical characteristics at dapagliflozin initiation and other medication prescribed.

	Value	95% CI
*Time between diagnosis and dapagliflozin initiation*, *n* = 220, months		
Mean	20.9	15.3–26.4
*HF-related hospitalization in 12 months prior to index*, *n* = 237, *n* (%)		
Yes	51.0 (21.5)	6.5 -27.3
*Time since last HF-related hospitalization*, *n* = 45, days		
Mean	109.5	77.9–141.1
*Principal cause of heart failure*, *n* = 237, *n* (%)		
Ischemic	66 (27.8)	22.2–34.0
Non-ischemic	73 (30.8)	25.0–37.1
Unknown	98 (41.4)	35.0–47.9
*NYHA class*, *n* = 237, *n* (%)		
Class I	43 (18.1)	13.5–23.7
Class II	117 (49.4)	42.8–55.9
Class III	42 (17.7)	13.1–23.2
Class IV	1 (0.4)	0.0–2.3
Unknown	34 (14.3)	10.1–19.5
*Left ventricular ejection fraction*, *n* = 182, %		
Mean	28.1	26.9–29.3
*Heart rate*, beats per minute, *n* = 227		
Mean	72.4	71.5–74.4
*Systolic blood pressure*, *n* = 224, mm Hg		
Mean	123.5	120.6–126.4
*Diastolic blood pressure*, *n* = 224, mm Hg		
Mean	72.6	70.7–74.5
*NT-proBNP level*, *n* = 86, pg/mL		
Mean	3335	2080–4591
*eGFR*, *n* = 218, mL/min/1.73m^2^		
Mean	64.6	62.3–66.9
*HbA1c*, *n* = 148, %		
Mean	8.7	7.1–10.4
*Comorbidities*^a^, *n* = 237, (%)		
Atrial fibrillation	77 (32.5)	26.6–38.9
Hypertension	70 (29.5)	23.8–35.8
Myocardial infarction	54 (22.8)	17.6–28.7
Chronic kidney disease	33 (13.9)	9.8–19.0
Diabetes mellitus type 2	32 (13.5)	9.4–18.5
Coronary revascularization	26 (11.0)	7.3–15.7
Other or none	53 (22.4)	17.2–28.2

95% CI, 95% confidence interval; ACE, angiotensin-converting enzyme; eGFR, eGFR; HbA1c, haemoglobin A1C; HF, heart failure; mm Hg, millimetres of mercury; NT-proBNP, N-terminal pro-B-type natriuretic peptide; NYHA, New York Heart Association.

a Reported for >10% of patients, comorbidities reported for <10% of patients include: significant heart valve regurgitation/stenosis; any stroke; cancer; implantable cardioverter-defibrillator; cardiac resynchronization therapy; psychiatric disorders e.g., depression, bipolar disorder, schizophrenia; unstable angina; myocarditis; dementia; HIV infection.

### Discontinuation

**Figure 1:**
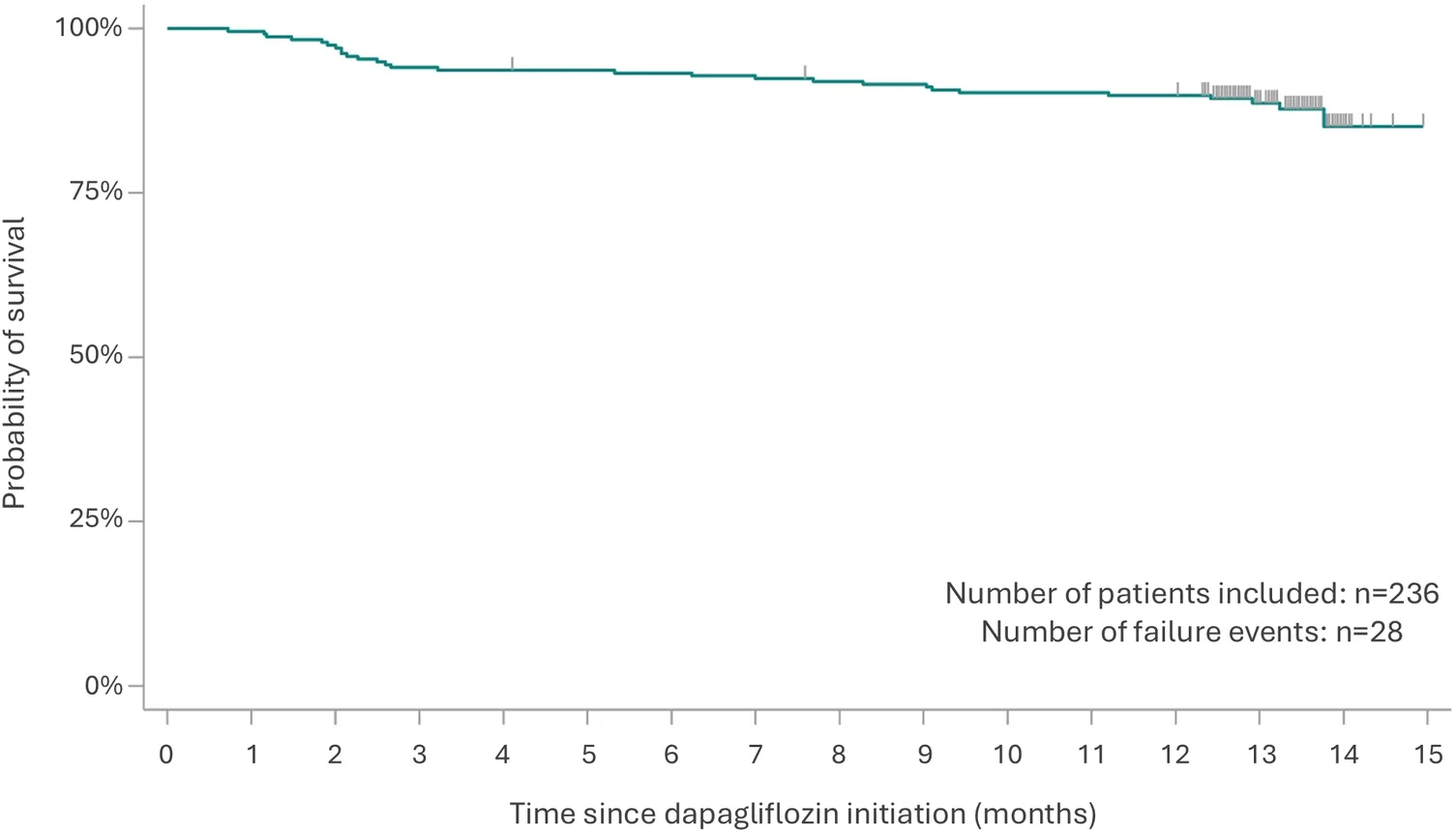
Kaplan meier plot of dapagliflozin discontinuations.

A Kaplan–Meier plot of discontinuations is shown in Figure 1. By the 3-month follow-up, 12 (5.1%) of patients had discontinued, 4 (1.7%) patients did so at each of the 6 and 9 months timepoints, and 1 (0.43%) further patient had discontinued treatment at 12 months. A total of 21 (8.9%) patients discontinued treatment during a total at-risk time of 87,515 days, leading to a discontinuation rate of 11.7/100 patient-years. N = 7 (3.0%) patients died within the 12-month observation period.

Reasons for discontinuation are shown in Table 3. The most common reason for discontinuation was an adverse drug reaction or adverse event, in 13 patients (61.9% of discontinuations), while 7 patients discontinued due to other relevant clinical event (33.3% of discontinuations). Cost and switch to another SGLT2i treatment were recorded for one discontinuation each, and in one case the reason for discontinuation was unknown.

**Table 3:** Reasons for discontinuation.

	Value	95% CI
*Reasons for discontinuation during study*^a^^,^ *n* = 21, *n* (%)		
Adverse drug reaction/adverse event	13 (61.9)	38.4–81.9
Cost	1 (4.8)	0.1–23.8
Switch to other SGLT2i treatment/combination	1 (4.8)	0.1–23.8
Other relevant clinical event	7 (33.3)	14.6–57.0
Unknown reason	1 (4.8)	0.1–23.8
*Reasons for discontinuation between enrolment and 3 months*^a^, *n* = 12, *n* (%)		
Adverse drug reaction/adverse event	8 (66.7)	34.9–90.1
Other relevant clinical event	4 (33.3)	9.9–65.1
Switch to other SGLT2i treatment/combination	1 (8.3)	0.2–38.5
*Reasons for discontinuation between 3 and 6 months*^a^, *n* = 4, *n* (%)		
Adverse drug reaction/adverse event	3 (75.0)	19.4–99.4
Cost	1 (25.0)	0.6–80.6
Other relevant clinical event	1 (25.0)	0.6–80.6
*Reasons for discontinuation between 6 and 9 months*^a^, *n* = 4, *n* (%)		
Adverse drug reaction/adverse event	2 (50.0)	6.8–93.2
Other relevant clinical event	1 (25.0)	0.6–80.6
Unknown reason	1 (25.0)	0.6–80.6
*Reasons for discontinuation between 9 and 12 months*^a^, *n* = 1, *n* (%)		
Other relevant clinical event	1 (100.0)	2.5–100.0

95% CI: 95% confidence interval.

a More than one reason could be selected by respondents.

### Concomitant prescribing

**Figure 2:**
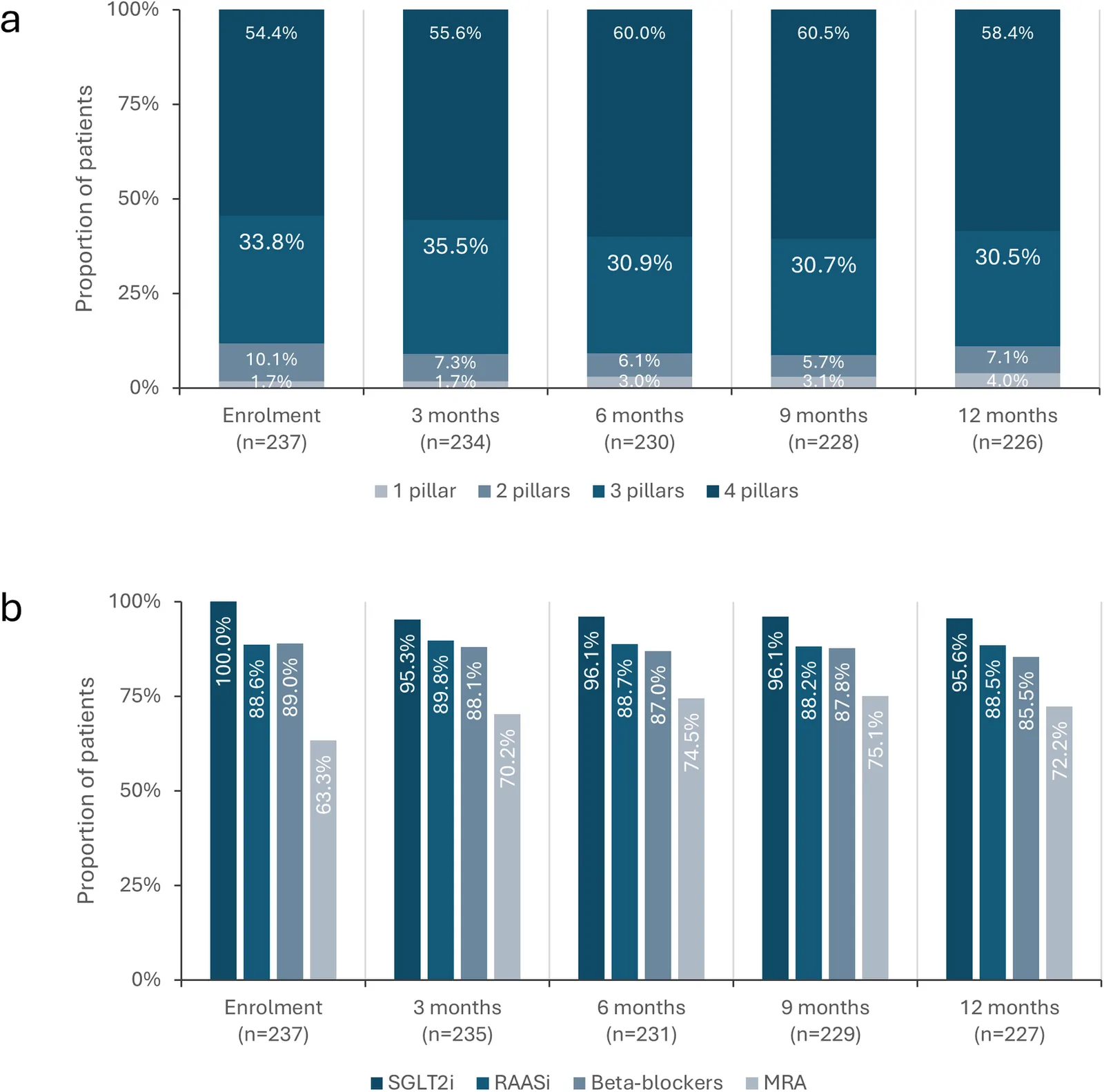
Concomitant treatment received by patients during the 12 months of follow-up. **(a)** Proportion of patients receiving HF treatment pillars. **(b)** Proportion of patients receiving treatment classes.

Following dapagliflozin initiation at index, 54.4% of patients were prescribed all four pillars of guideline-recommended HF treatment, while a further 33.8% were prescribed three pillars (Figure 2a). After 12 months of follow-up, 58.4% of patients were receiving four pillars and 30.5% were receiving three. Over the follow-up period, SGLT2i use ranged from 100.0% to 95.3% of patients, RAASi use between 88.2% and 89.8%, beta blocker use between 85.5% and 89.0%, and MRA use between 63.3% and 75.1% (Figure 2b). Within the RAASi pillar, patients were most commonly prescribed an angiotensin receptor-neprilysin inhibitor (ranging from 54.4% to 67.4% during the follow-up period), followed by an angiotensin converting enzyme inhibitor (ranging from 16.3% to 27.8%) or an angiotensin receptor blocker (ranging from 4.8% to 8.9%).

The specific treatment pillar combinations prescribed alongside an SGLT2i are shown in Supplementary Figure 2. The most common three-pillar treatment combination with a SGLT2i prescribed at all timepoints was an SGLT2i with a beta blocker and a RAASi (27.0% of patients at enrolment, 18.6% at 12 months).

Changes between treatment pillars during the follow-up period are shown in Supplementary Figure 3. Most changes occurred between enrolment and 3 months or 3 months and 6 months. Prior to the 3-month timepoint, patients most commonly changed from three to four pillars or four to three pillars (6.3% of patients each). Between 3 and 6 months, 6.8% of patients changed from three to four pillars and 3.0% changed from four to three pillars.

### Patient-reported KCCQ-23 scores

**Figure 3:**
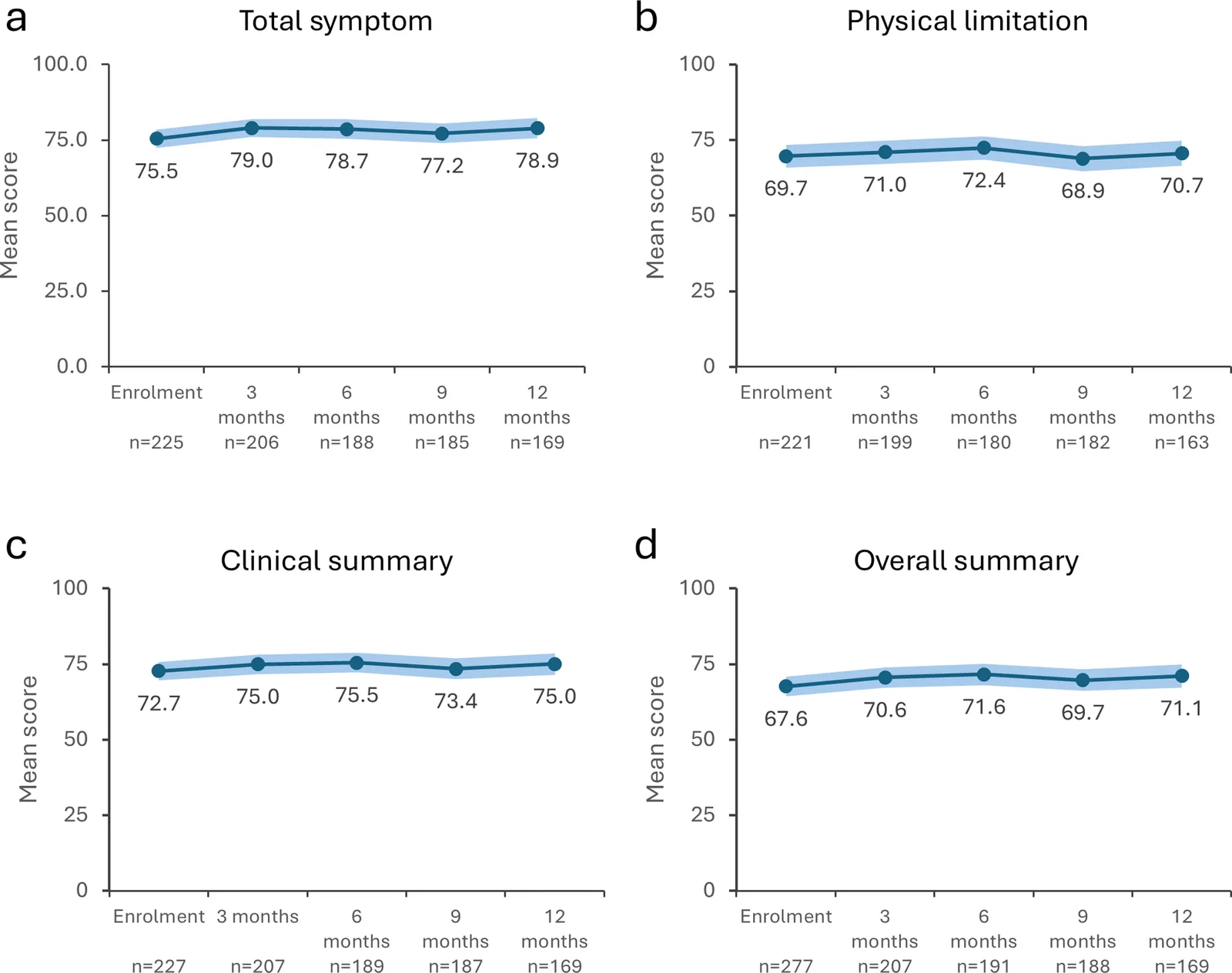
Mean (95% CI) KCCQ scores per domain with 95% confidence interval, over time. **(a)** Total symptom score. **(b)** Physical limitation score. **(c)** Clinical summary score. **(d)** Overall summary score.

The mean (95% confidence interval) total symptom score reported by patients ranged between 75.5 (72.4–78.5) at enrolment to 79.0 (76.0–82.0) at 3 months, reaching 78.9 (75.6–82.3) at 12 months, Figure 3a. Mean physical limitation score at enrolment was 69.7 (66.0–73.5), and ranged between 72.4 (68.6–76.2) at 6 months and 68.9 (64.8–73.0) at the 9-month follow-up, before reaching 70.7 (66.4–74.9) at 12 months (Figure 3b).

The clinical summary score was 72.7 (69.6–75.8), 75.5 (72.2–78.8), and 75.0 (71.5–78.6) at enrolment, 6 and 12 months respectively (Figure 3c). The overall summary score was 67.6 (64.3–70.9), and 71.6 (68.1–75.1) and 71.1 (67.3–74.9) at enrolment, 6 and 12 months respectively (Figure 3d).

### Patient-reported treatment adherence

**Figure 4:**
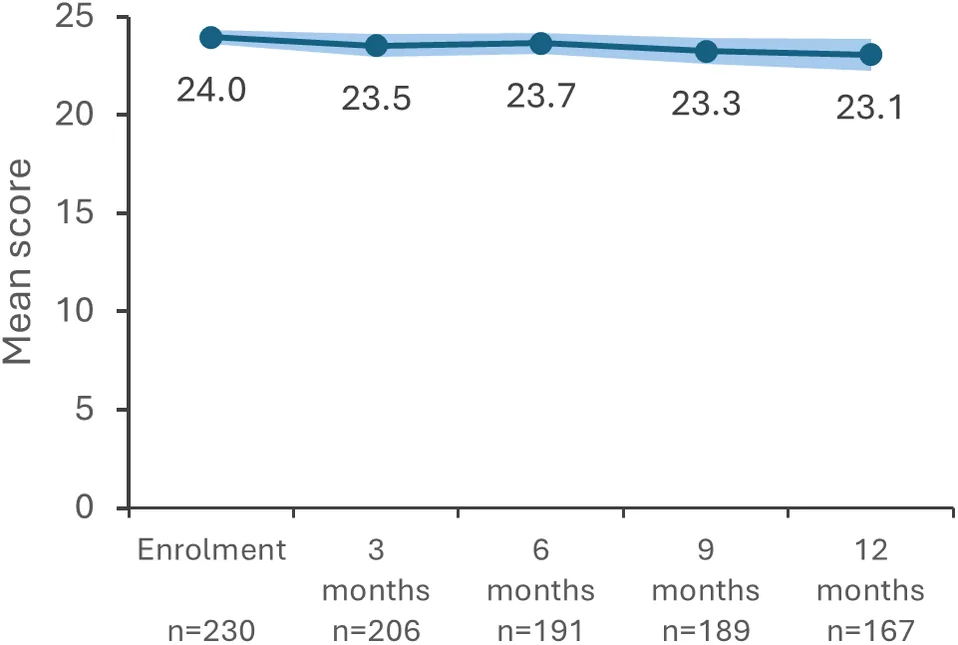
Mean (95% CI) MARS-5 scores over time with 95% confidence interval.

Mean (95% CI) MARS-5 scores, representing patient-reported treatment adherence, including to dapagliflozin, ranged from 24.0 (23.6–24.3) at enrolment to 23.1 (22.3–23.9) out of a possible 25 at 12 months (Figure 4).

### Patient-reported work and activity impairment

**Figure 5:**
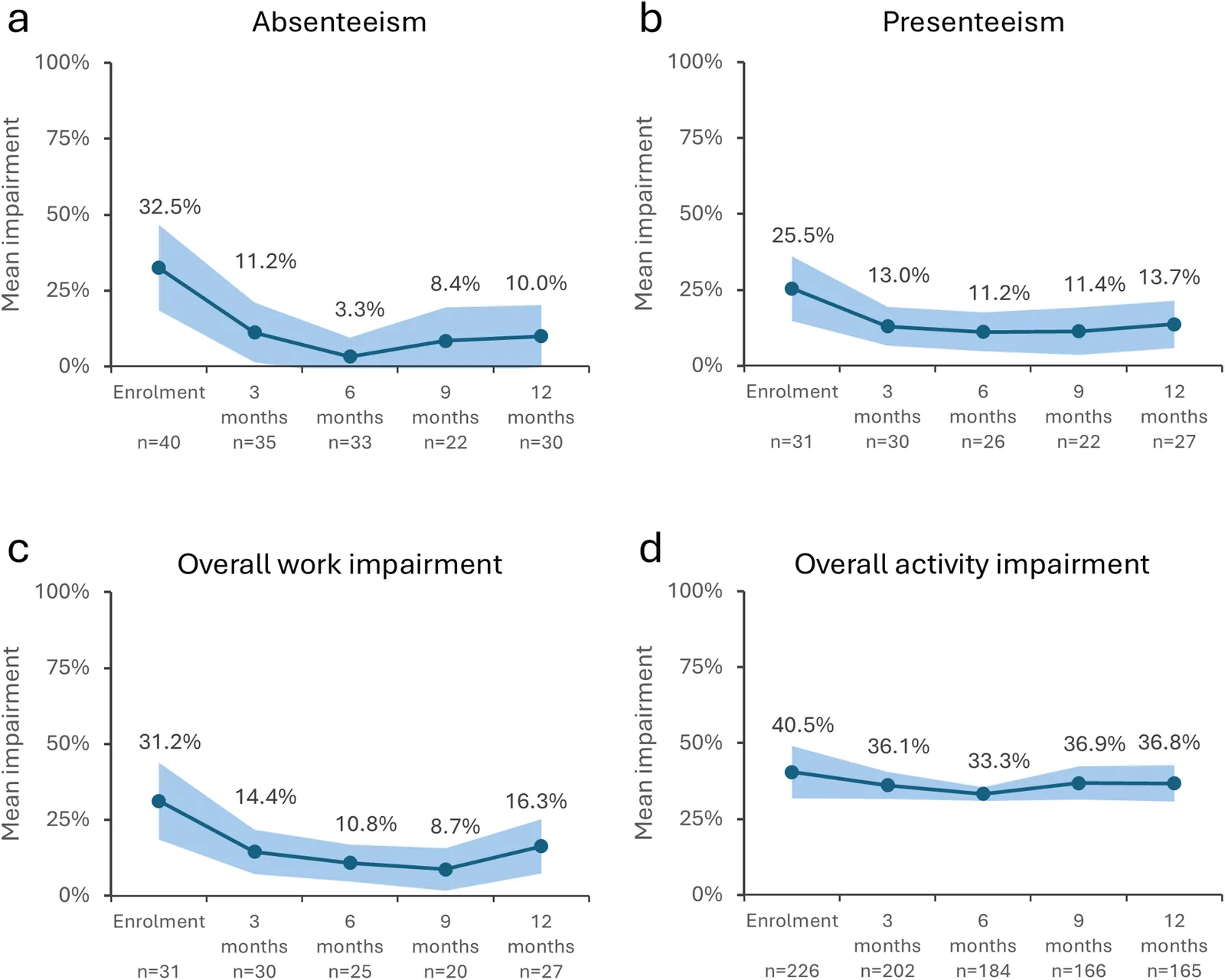
WPAI impairment per domain with 95% confidence interval, over time. **(a)** Absenteeism. **(b)** Presenteeism. **(c)** Overall work impairment. **(d)** Overall activity impairment.

Mean (95% CI) patient-reported levels of absenteeism in the WPAI ranged from 32.5% (18.4–46.6%) at enrolment, to 3.3% (−2.9%–9.5%) at 6 months, reaching 10.0% (−0.3–20.3%) at 12 months (Figure 5a), while presenteeism ranged from 25.5% (14.8–36.2%) at enrolment, to 11.2% (4.8–17.5%) at 6 months, reaching 13.7% (5.9–21.5%) at 12 months (Figure 5b). Overall work impairment ranged from 31.2% (18.5–43.9%) at enrolment, to 8.7% (1.7–15.6%) at 9 months, and was 16.3% (7.4–25.2%) at 12 months (Figure 5c). Overall activity impairment, including patients not in employment, ranged from 40.5% (36.3–44.7%) at enrolment to 33.3% (28.8–37.8%) at 6 months, and was 36.8% (32.1–41.5%) at 12 months (Figure 5d**)**.

## Discussion

Dapagliflozin has been shown to reduce mortality and hospitalization in HFrEF in clinical trials, but there is limited information about its use in real-world UK clinical practice. In this study, we used a multi-centre longitudinal study design to investigate demographic and clinical characteristics of patients prescribed dapagliflozin, prescribing patterns and patient QoL in the 12 months following initiation of dapagliflozin.

Patients had a relatively long time between diagnosis and dapagliflozin initiation. As the study took place soon after the UK approval dapagliflozin in the UK, which took place in February of 2021 (7), reported times between diagnosis and dapagliflozin initiation are likely biased towards being longer due to patients being diagnosed prior to approval. Furthermore, it is known from data in the United States that factors such as clinical inertia, lack of familiarity, knowledge, and comfort with use, and concerns over polypharmacy or adverse events may be barriers to prescribing SGLT2i (12), which may delay initiation. Further research would be needed to determine if this was the case in the UK as well.

Numbers of patients discontinuing dapagliflozin were low, at 8.9% 12 months post-initiation. Although comparing discontinuation rates is difficult, due to possible confounding differences in patient populations and different analysis windows, this discontinuation rate compares favourably to others reported in real-world HFrEF practice. In a review of UK HFrEF care, 13.0% of patients discontinued dapagliflozin in seven months of treatment (13), whereas 23.5% of patients discontinued at one year in a multinational study (14). In a trial for dapagliflozin in HF with preserved ejection fraction, 14.3% discontinued at a median follow-up time of 2.3 years (15).

The comparatively low discontinuation rate for dapagliflozin in our cohort suggests it is well tolerated in this patient population. The most common reason for discontinuation in this study was an adverse drug reaction or other adverse event, in line with previously reported reasons for SGLT2i discontinuation in diabetes care (16, 17).

Almost half of patients did not receive all four guideline-recommended treatment pillars. In comparison, studies across Europe, the US and Japan report less than 20% of patients received all four pillars (18, 19). However, our data are biased due to the eligibility requirements of receiving dapagliflozin. Indeed, one US study reported the proportion of patients receiving all four treatment pillars specifically in those who were prescribed an SGLT2i was 46.3% (20), largely comparable to our cohort. The treatment class most commonly omitted from patients’ treatment in our cohort was an MRA, in line with previously reported low prescription rates of MRAs in HFrEF (18, 19).

Although our data suggest a modest increase in the number of patients receiving four pillars of treatment during the follow-up period, it remains evident that a significant proportion of patients did not receive maximal guideline-recommended treatment at any time during our study. Clinical inertia, where patients do not receive optimal management as defined by treatment guidelines, has been recognized as a major issue in HF by the European Society of Cardiology (21).

A number of reasons have been suggested in the literature for physicians not prescribing according to guidelines, including concerns surrounding hypotension, kidney function, hyperkalaemia and polypharmacy (21), as well as patient-related factors, such as lack of adherence, and patient preferences and beliefs (22). Given observed blood pressure and kidney function in this cohort, it is unlikely that physiological concerns drove any suboptimal treatment, as rapid four pillar treatment optimization was likely clinically feasible. However, physicians did alter treatment after dapagliflozin initiation, indicating possible room for optimization of treatment in patients with established HF.

Applying commonly used thresholds (23), patients’ recorded KCCQ scores were in the fair-to-good range (physical limitation, clinical summary and overall summary scores) or good-to-excellent range (total symptom scores) throughout the study period, staying within the 5-point range that is considered a minimal important difference (23). Our results indicate that patients perceived their disease as relatively stable during the study period. A significant effect of dapagliflozin on multiple KCCQ domains was previously noted during clinical trials, where patients saw a significant improvement over placebo (24, 25), however, few data were available from real-world clinical practice prior to this study.

Patients recorded high MARS-5 scores throughout the study, suggesting good adherence to their overall HF treatment, including dapagliflozin. This is somewhat in contrast to previous reports in type 2 diabetes, where adherence was found to be suboptimal (26), and adherence for dapagliflozin was worse than for other SGLT2is (27). The relatively high levels of patient-reported adherence observed here support good tolerability of dapagliflozin in this HFrEF population.

Patients reported notable levels of impairment in the WPAI at initiation of dapagliflozin, with mean absenteeism, overall work impairment, and overall activity impairment above 30%. This is in line with previous reports from Europe and China (28–30). Given that mean time between diagnosis of HFrEF and dapagliflozin initiation was almost two years, this impairment level is unlikely due to being newly diagnosed and untreated. To date, the effect of dapagliflozin treatment on WPAI measures is unknown, but our data suggest a potential beneficial effect.

### Strengths and limitations

A strength of the study is the broad geographical spread of enrolled patients across the UK. However, the study cohort was predominantly male and White, limiting the representativeness of the cohort in female and ethnic minority patients. Furthermore, most patients in this cohort had NYHA class II disease with relatively preserved renal function, limiting representativeness of patients with more severe disease. We also cannot completely exclude selection bias for both participating centres and patients enrolled. Interpretation of the data may be confounded by the fact that enrolment took place at least 14 days following dapagliflozin initiation, meaning some early effects may have been missed, and by the simultaneous optimization of other treatments that took place during the study period. The study did not have a control arm, which limits interpretability of the results, as we cannot distinguish if any effects seen are due to dapagliflozin or other treatment received or the potential impact of natural HF progression during the study period.

## Conclusion

In conclusion, this study showed fair-to-good quality of life amongst treated patients receiving dapagliflozin as part of their HFrEF treatment. It also demonstrated low discontinuation rates after the initiation of dapagliflozin and good adherence. Furthermore, it highlights an opportunity for further optimization of guideline-directed medical therapy to improve outcomes in patients with HFrEF.

## Data Availability

Data underlying the findings described in this article may be obtained in accordance with AstraZeneca's data sharing policy described at https://astrazenecagrouptrials.pharmacm.com/ST/Submission/Disclosure. Data for studies directly listed on Vivli can be requested through Vivli at www.vivli.org. Data for studies not listed on Vivli could be requested through Vivli at https://vivli.org/members/enquiries-about-studies-not-listed-on-the-vivli-platform/. AstraZeneca Vivli member page is also available outlining further details: https://vivli.org/ourmember/astrazeneca/. Further inquiries should be directed to the corresponding author.
