# Supplementary material for: Observational study on initiation of dapagliflozin in heart failure with reduced ejection fraction in the United Kingdom
Source: Front Cardiovasc Med. 2026 Sep 8;13:1858743. doi: 10.3389/fcvm.2026.1858743 (PMC13593797; doi:10.3389/fcvm.2026.1858743)
Supplement: Supplementary file 1 [file Supplementaryfile1.docx]

## Supplementary material

**Supplementary table 1** – Hospital centres included in the study

University Hospitals Sussex NHS Foundation Trust

Northern Devon Healthcare NHS Trust

Royal Cornwall Hospitals NHS Trust

University Hospitals of North Midlands NHS Trust

University Hospitals Coventry and Warwickshire NHS Trust

Mid and South Essex NHS Foundation Trust

Southern Health and Social Care Trust

Royal Berkshire NHS Foundation Trust

Swansea Bay University Local Health Board

King's College Hospital NHS Foundation Trust

**Supplementary figure 1** – Study design and attrition numbers. Patient response rates varied between PROMs, the highest number of responses at each timepoint is reported.

**Supplementary Figure 2** – Treatment received within treatment pillars alongside SGLT2i.

**Supplementary Figure 3** – Changes between treatment pillars during the 12 month follow-up
